# Gestational weight gain adequacy among twin pregnancies in France

**DOI:** 10.1111/mcn.13436

**Published:** 2022-10-12

**Authors:** Melissa Amyx, Diane Korb, Jennifer Zeitlin, Thomas Schmitz, Camille Le Ray

**Affiliations:** ^1^ Obstetrical Perinatal and Pediatric Epidemiology Research Team (EPOPé), Center for Research on Epidemiology and Statistics Sorbonne Paris Cité (CRESS), INSERM, INRA Université de Paris Cité Paris France; ^2^ Service de Gynécologie Obstétrique, Hôpital Robert Debré, Assistance Publique‐Hôpitaux de Paris Université de Paris Cité Paris France; ^3^ Maternité Port Royal, Hôpital Cochin Port Royal, Assistance Publique‐Hôpitaux de Paris Université de Paris Cité Paris France

**Keywords:** body mass index, classification, gestational weight gain, Institute of Medicine, pregnancy, pregnancy weight gain, twins

## Abstract

The objective of this paper is to describe gestational weight gain (GWG), to assess the applicability of the 2009 Institute of Medicine (IOM) guidelines, and to derive a GWG adequacy classification within a French cohort. We included twins from the national, prospective, population‐based JUmeaux MODe d'Accouchement (JUMODA) cohort study (2014–2015). Following the IOM approach, we selected a ‘standard’ population of term pregnancies with ‘optimal’ birthweight (≥2500 *g*; *n* = 2562). GWG adequacy (insufficient; adequate; excessive) was defined using IOM recommendations (normal body mass index [BMI]: 16.8–24.5 kg [also utilized for underweight BMI]; overweight: 14.1–22.7 kg; obese: 11.4–19.1 kg). Additionally, using the IOM approach, we determined the 25th and 75th percentiles of GWG in our standard population to create a JUMODA‐derived GWG adequacy classification. GWG and GWG adequacy were described, overall and by BMI and parity. In the JUMODA standard population of term twin livebirths with optimal birthweight, mean GWG was 16.1 kg (standard deviation 6.3). Using IOM recommendations, almost half (46.5%) of the women had insufficient and few (10.0%) had excessive GWG, with similar results regardless of BMI or parity. The 25th and 75th percentiles of GWG in the JUMODA standard population (underweight: 13–21 kg; normal weight: 13–20 kg; overweight: 11–19 kg; obese: 7–16 kg) were lower than the IOM recommendations. The IOM recommendations classified a relatively high percentage of French women as having insufficient and a low percentage as having excessive GWG. Additional research to evaluate recommendations in relation to adverse perinatal outcomes is needed to determine whether the IOM recommendations or the JUMODA‐derived classification is more appropriate for French twin gestations.

## Key messages

- Using the 2009 Institute of Medicine (IOM) recommendations to define gestational weight gain (GWG) adequacy in our French cohort classified almost half of women as having insufficient GWG and a relatively low percentage as having excessive GWG.
- The USA‐derived IOM definition for adequate GWG may not apply in France or in other non‐USA, contemporary obstetric populations.
- Additional research in large, population‐based contemporary cohorts with prospective GWG ascertainment and assessment of GWG adequacy classifications in relation to adverse outcomes is needed to inform evidence‐based GWG recommendations for twin pregnancies.

## Graphical abstract

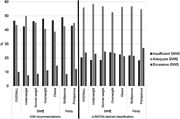

## 1 INTRODUCTION

In 2009, the United States Institute of Medicine (IOM) released updated guidelines for gestational weight gain (GWG), with specific guidelines added for twin pregnancies (Rasmussen & Yaktine, 2009). The provision of guidelines for twin pregnancies was an important step given the association between adverse outcomes and both insufficient (e.g., preterm delivery [Lipworth et al., 2022; Zhong et al., 2021], gestational diabetes mellitus, low birthweight, neonatal intensive care unit admission [Zhong et al., 2021]) and excessive (e.g., Pre‐eclampsia [Lipworth et al., 2022], gestational hypertension, caesarean section, preterm birth [Zhong et al., 2021]) GWG in twin pregnancies. Furthermore, compared to singleton pregnancies, twin pregnancies have a higher incidence of poor obstetric and perinatal outcomes associated with insufficient GWG (e.g., foetal and neonatal death, small for gestational age [GA]) and of increased GWG and pregnancy complications associated with excessive GWG (e.g., Pre‐eclampsia, gestational diabetes, caesarean section [Bodnar et al., 2014; Hutcheon et al., 2018; Widen, 2018]).

Nonetheless, the IOM acknowledged that the guidelines for GWG in twin pregnancies were provisional because the committee was not able to evaluate the causal association between GWG and adverse outcomes and the trade‐offs between maternal and child outcomes, as was done for the singleton GWG recommendations. Instead, the IOM recommendations for twin pregnancies were based on the 25th and 75th percentiles of GWG in a historical USA cohort (1979–1999) of term (37–42 weeks GA) livebirths with optimal birthweight (mean twin birthweight of ≥2500 *g* [Luke et al., 2003]) and due to small sample size, recommendations for underweight women were not provided (Rasmussen & Yaktine, 2009).

Given the IOM recommendations were not intended for use outside the USA (particularly outside developed countries, in populations with shorter or thinner women, or where adequate obstetric services are unavailable [Rasmussen & Yaktine, 2009]), verification of their applicability in specific obstetric populations and estimation of potential cohort‐derived percentiles is important to ensure pregnant women receive appropriate GWG counselling. Nonetheless, few studies, and none in France, have applied the IOM methodology in contemporary, non‐USA standard populations to verify similarity of GWG patterns. While evidence in France is lacking, appropriate GWG in twins is a pertinent topic as twin gestations (2010: 2.9%; 2016: 3.4%) and maternal prepregnancy body mass index (BMI) are increasing (overweight, 2010: 17%; 2016: 20%; obese, 2010: 10%; 2016: 12% [Coulm et al., 2017]). However, the French obstetric population (underweight: 7.4%; normal weight 60.8%; overweight: 20.0%; obese: 11.8% [Coulm et al., 2017]) is thinner that than in the United States (underweight: 3.6%; normal weight 45.0%; overweight 25.8; obese 25.6% [Deputy et al., 2018]), which the IOM noted may impact their recommendations' applicability (Rasmussen & Yaktine, 2009). Indeed, a previous study in French liveborn twins delivered after 23 weeks 6 days (nonstandard population including all GAs, birthweights) found that the IOM guidelines classified a high percentage of twin pregnancies as having insufficient and a low percentage as having excessive GWG (Pécheux et al., 2019).

Therefore, the objectives of this study were to describe GWG, assess the applicability of the 2009 IOM GWG guidelines, and derive a GWG adequacy classification utilizing the IOM methodology within a French standard population derived from a contemporary, national, population‐based French cohort.

## 2 METHODS

### 2.1 Source population

We used data from the JUmeaux MODe d'Accouchement (JUMODA) study (February 10, 2014–March 1, 2015), a national, prospective, population‐based cohort study including women giving birth to twins ≥22 weeks GA. All French maternity units (academic, public, private hospitals; regardless of level of care) with over 1500 deliveries in France were eligible (176/191 eligible units [92%] agreed [Schmitz et al., 2017]). Participants received information and provided oral informed consent to participate before recruitment and data collection.

### 2.2 Study population

Of 8823 women recruited immediately following delivery into the initial JUMODA cohort (Supporting Information: Figure 1), we excluded women not delivering two liveborn twins (*n* = 320), as pregnancies with one or more stillbirth may have distinct GWG patterns, or with missing (*n* = 1303) or implausible GWG (>50 kg; *n* = 3). Then, we excluded women with preterm birth (before 37 weeks GA; *n* = 3732), missing BMI (*n* = 66; GWG adequacy determination not possible), or with mean twin birthweight <2500 g (*n* = 4109) to create a standard population of twin pregnancies (*n* = 2562) equivalent to that utilized to derive the IOM guidelines (term livebirths with optimal birthweight). Data utilized were collected by trained research nurses through chart abstraction.

### 2.3 Variables

GWG was determined based on maternal end of pregnancy minus beginning of pregnancy weight (kg). GWG adequacy by maternal prepregnancy BMI (underweight < 18.5; normal weight 18.5–24.9; overweight 25–29.9; obese ≥30 kg/m^2^; calculated using maternal height and beginning of pregnancy weight) was initially defined using the 2009 IOM recommendations (normal weight: 16.8–24.5 kg, also applied to underweight BMI group; overweight: 14.1–22.7 kg; obese: 11.4–19.1 kg [Rasmussen & Yaktine, 2009]). GWG adequacy was additionally defined using a JUMODA‐derived GWG adequacy classification (outlined below). For both the IOM and JUMODA‐derived GWG adequacy classifications, GWG adequacy was defined as: insufficient (below 25th percentile), adequate (25th–75th percentile), or excessive (above 75th percentile).

Stratification variables included pre‐pregnancy BMI (defined above) and GA at birth (completed weeks; ≥39 weeks combined 39–41 weeks GA as French guidelines recommend delivery before 40 weeks for dichorionic and before 39 weeks for monochorionic twins [Vayssière et al., 2011] and thus few women with twin pregnancies in France reach 40 weeks [and none in our cohort reached 42 weeks GA]). Maternal characteristics utilized to describe the standard population included: GA at delivery (days), parity (nullipara; multipara), age (<30; 30–34; ≥35 years), country or region of birth (France; Europe; Northern Africa; Africa others; other), smoking during pregnancy (yes; no), profession (managers and higher intellectual professions; intermediate professions, employees; craftsmen, storekeepers; farmers, workers; retired, nonworking), assisted reproductive technology use (none; in vitro fertilization or intracytoplasmic sperm injection), Pre‐eclampsia (yes; no), diabetes or gestational diabetes (yes; no), intrauterine growth restriction of either twin (yes; no), suspicion of macrosomia of either twin (yes; no), and chorionicity (dichorial; monochorial; missing).

### 2.4 Statistical analysis

Maternal characteristics and GWG of the JUMODA standard population were described. GWG was described overall and by GA at delivery (means, standard deviations [SDs], 25th and 75th percentiles), with further stratification by BMI.

GWG adequacy based on the IOM recommendations was determined in the JUMODA standard population to assess their applicability in a French cohort similar to that in which they were derived (in terms of GA at delivery and twin birthweight). Then, we created a JUMODA‐cohort derived GWG adequacy classification by utilizing the approach the IOM used to generate their recommendations: we determined the 25th and 75th percentiles of total GWG, by prepregnancy BMI, within our standard population (women delivering twin livebirths at 37–42 weeks GA with optimal birthweight [≥2500 *g*]). GWG adequacy based on the JUMODA‐derived classification, overall and by prepregnancy BMI and parity, was determined in the standard population to verify the performance of the classification. Additionally, in line with the IOM report, adjusted cumulative GWG was presented as least square means (standard error) from multivariable linear regression models adjusted for smoking in pregnancy, parity, chorionicity, Pre‐eclampsia, diabetes or gestational diabetes, and GA at delivery (days).

We used SAS software version 9.4 for Windows (SAS Institute Inc.) for statistical analyses.

### 2.5 Ethical statement

Women received information and provided oral informed consent for study participation before recruitment and data collection. JUMODA was approved by the National Data Protection Authority, the Consultative Committee on the Treatment of Information on Personal Health Data for Research Purposes, and the Committee for the Protection of People Participating in Biomedical Research before the study began.

## 3 RESULTS

**Figure 1:**
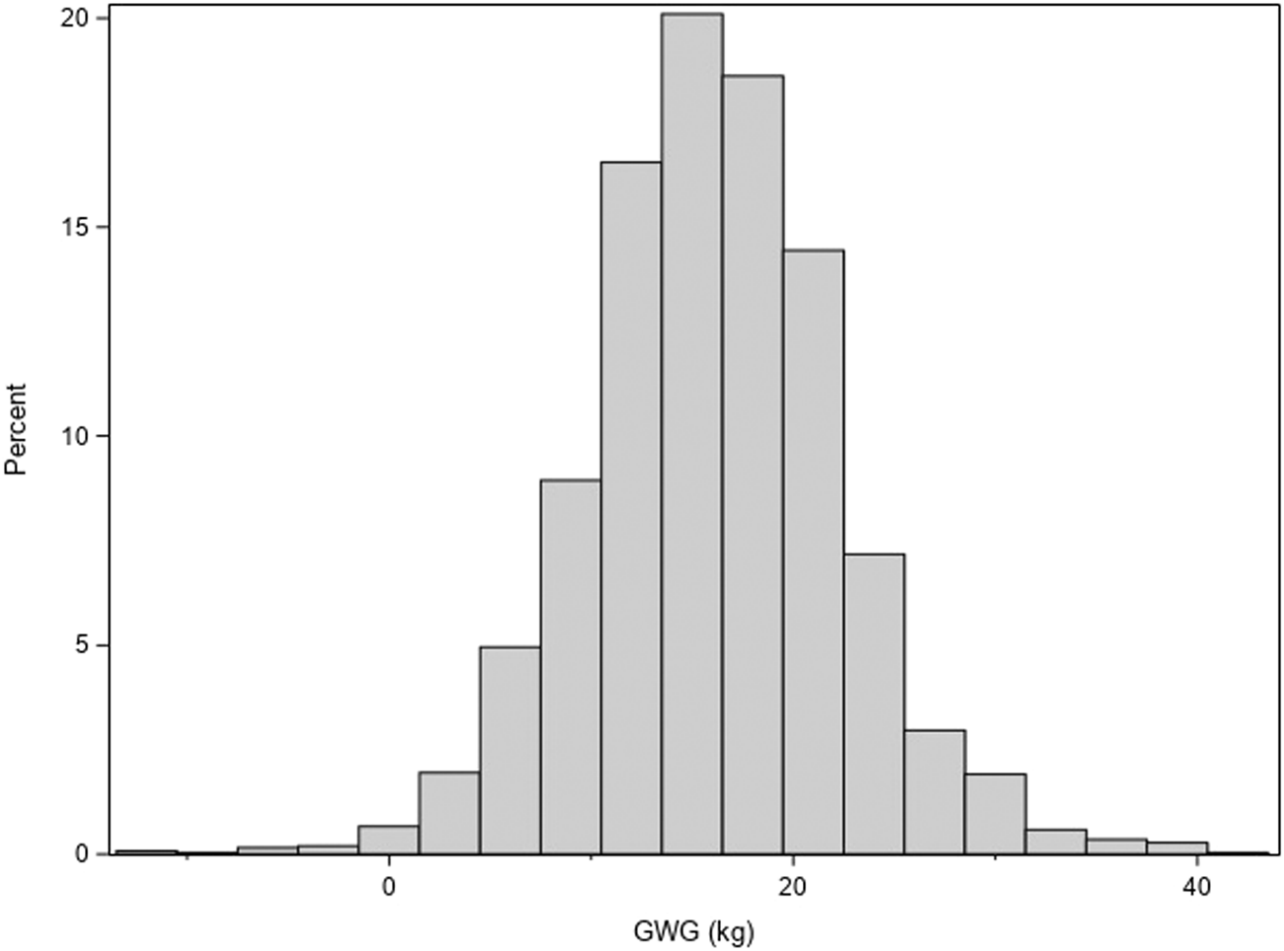
Distribution of GWG (kg) in the JUMODA standard population (*N* = 2562) of term (37–42 weeks gestational age) twin livebirths with mean twin birthweight ≥2500 *g* (France, 2014–2015). GWG, gestational weight gain

In the JUMODA standard population of term twin livebirths with optimal birthweight, most women were normal weight prepregnancy BMI (59.9%), were born in France (75.6%), and did not smoke in pregnancy (90.4%; Table 1). Mean cumulative GWG was 16.1 kg (SD 6.3; 25th, 75th percentiles of total GWG 12, 20 kg) and increased from 37 to ≥39 weeks (Table 2; Figure 1). GWG was inversely related to prepregnancy BMI, but patterns were inconsistent across BMI strata for the association between GWG and GA at delivery.

**Table 1:** Maternal and pregnancy characteristics in a standard population of liveborn term births (37–42 weeks gestation) with mean birthweight ≥2500 *g* (JUMODA cohort, 2014–2015; France)

	Study population
	*N* = 2562
	*n* (%)
Gestational age at delivery (days), mean (SD)	267.2 (4.9)
BMI,^a^ mean (SD)	24.5 (5.1)
Underweight	118 (4.6)
Normal weight	1534 (59.9)
Overweight	562 (21.9)
Obese	348 (13.6)
Parity	
Nullipara	1041 (40.7)
Multipara	1518 (59.3)
Age (years)	
<30	808 (31.5)
30–34	967 (37.7)
≥35	787 (30.7)
Country or region of birth	
France	1747 (75.6)
Europe	94 (4.1)
Northern Africa	301 (13.0)
Africa others	133 (5.8)
Other	35 (1.5)
Smoking during pregnancy	
No	2238 (90.4)
Yes	237 (9.6)
Profession	
Managers and higher intellectual professions	425 (17.9)
Intermediate professions, employees	1232 (51.9)
Craftsmen, storekeepers	80 (3.4)
Farmers, workers	41 (1.7)
Retired, nonworking	597 (25.1)
ART	
None	1976 (77.4)
IVF/ICSI	576 (22.6)
Pre‐eclampsia	
No	2443 (95.4)
Yes	119 (4.6)
Diabetes or gestational diabetes	
No	2240 (87.5)
Yes	321 (12.5)
IUGR (either twin)	
No	2448 (95.6)
Yes	114 (4.4)
Suspicion macrosomia (either twin)	
No	2099 (92.0)
Yes	183 (8.0)
Chorionicity	
Dichorial	2269 (88.6)
Monochorial	279 (10.9)
Missing	14 (0.5)

Abbreviations: ART, assisted reproductive technology; BMI, body mass index; ICSI, intracytoplasmic sperm injection; IUGR, intrauterine growth restriction; IVF, in vitro fertilization; SD, standard deviation.

^a^ BMI (kg/m^2^): underweight: <18.5; normal weight: 18.5–24.9; overweight: 25–29.9; obese: ≥30.

**Table 2:** GWG (kg) in a standard population of liveborn twin term births (37–42 weeks gestation) with mean birthweight ≥2500 *g* (JUMODA cohort, 2014–2015; France) by gestational age at delivery and prepregnancy BMI^a^

	*n*	Mean (SD)	25th, 75th percentiles
Overall			
37 weeks	926	15.8 (6.3)	12, 20
38 weeks	1220	16.1 (6.5)	12, 20
≥39 weeks^b^	416	16.4 (6.1)	12, 20
Underweight			
37 weeks	33	18.8 (5.4)	16, 22
38 weeks	59	17.8 (5.3)	13, 21
≥39 weeks^b^	26	16.6 (6.2)	13, 21
Normal weight			
37 weeks	542	17.1 (5.8)	13, 20
38 weeks	740	17.3 (5.5)	14, 20.75
≥39 weeks^b^	252	17.1 (5.3)	13, 20
Overweight			
37 weeks	214	14.4 (6.2)	10, 17
38 weeks	254	15.4 (6.7)	11, 20
≥39 weeks^b^	94	15.9 (6.0)	12, 20
Obese			
37 weeks	137	12.6 (6.8)	8, 16
38 weeks	167	11.2 (7.6)	6, 15
≥39 weeks^b^	44	13.1 (9.1)	7.5, 18

Abbreviations: BMI, body mass index; GWG, gestational weight gain; SD, standard deviation.

^a^ Prepregnancy BMI (kg/m^2^): underweight: <18.5; normal weight: 18.5–24.9; overweight: 25–29.9; obese.

^b^ Within this group, twenty‐three women delivered at/after 40 weeks gestations (1 underweight BMI; 17 normal weight BMI; 2 overweight BMI; 3 obese BMI).

Table 3 presents the IOM recommendations and the JUMODA‐derived GWG adequacy classification. Mean GWG was lower in the JUMODA standard population than in the IOM cohort, though mean GWG in JUMODA increased after adjustment. The 25th and 75th percentiles of total GWG in the JUMODA standard population (normal weight: 13–20 kg; overweight: 11–19 kg; obese: 7–16 kg) were lower than the IOM recommendations (normal weight: 16.8–24.5 kg; overweight: 14.1–22.7 kg; obese: 11.4–19.1 kg). While the IOM had insufficient underweight women to provide recommendations for this group, in the JUMODA standard population, the 25th, 75th percentiles of total GWG were 13, 21 kg (*n* = 118).

**Table 3:** GWG adequacy^a^ in twin pregnancies: The 2009 IOM recommendations and a JUMODA‐derived classification

Prepregnancy BMI^b^	IOM recommendations^c^				JUMODA^d^			
	*n*	Unadjusted mean (SD), kg	Adjusted mean (SE)^e^, kg	25th, 75th percentiles, kg	*n*	Unadjusted mean (SD), kg	Adjusted mean (SE)^f^, kg	25th, 75th percentiles, kg
Underweight	—	—	—	—	118	17.8 (5.5)	18.3 (0.9)	13, 21
Normal weight	409	21.0 (6.1)	20.9 (0.3)	16.8, 24.5	1534	17.2 (5.6)	17.7 (0.7)	13, 20
Overweight	154	18.7 (7.0)	18.9 (0.5)	14.1, 22.7	562	15.1 (6.4)	15.7 (0.7)	11, 19
Obese	143	15.4 (7.2)	15.7 (0.5)	11.4, 19.1	348	12.0 (7.5)	12.9 (0.7)	7, 16

Abbreviations: BMI, body mass index; GWG, gestational weight gain; IOM, Institute of Medicine; JUMODA, JUmeaux MODe d'Accouchement; SD, standard deviation; SE, standard error.

^a^ GWG considered adequate within the 25th, 75th percentiles.

^b^ BMI (kg/m^2^): underweight: <18.5; normal weight: 18.5–24.9; overweight: 25–29.9; obese: ≥30.

^c^ Derived from a historical US cohort (1979–1999) of term (37–42 weeks gestational age) twin livebirths with mean twin birthweight of ≥2500 *g* from four hospitals; separate recommendation for underweight women not provided due to small sample size.

^d^ JUMODA standard population of term (37–42 weeks gestational age) twin livebirths with mean twin birthweight ≥2500 *g* (2014–2015; France).

^e^ Values for least square means (SE mean) from models adjusted for diabetes and gestational diabetes, Pre‐eclampsia, smoking during pregnancy, primiparity, placental membranes (monochorionicity and missing chorionicity) and length of gestation.

^f^ Values for least square means (SE mean) from multivariable linear regression models adjusted for smoking in pregnancy, parity, chorionicity, Pre‐eclampsia, diabetes or gestational diabetes and gestational age at delivery (days).

**Figure 2:**
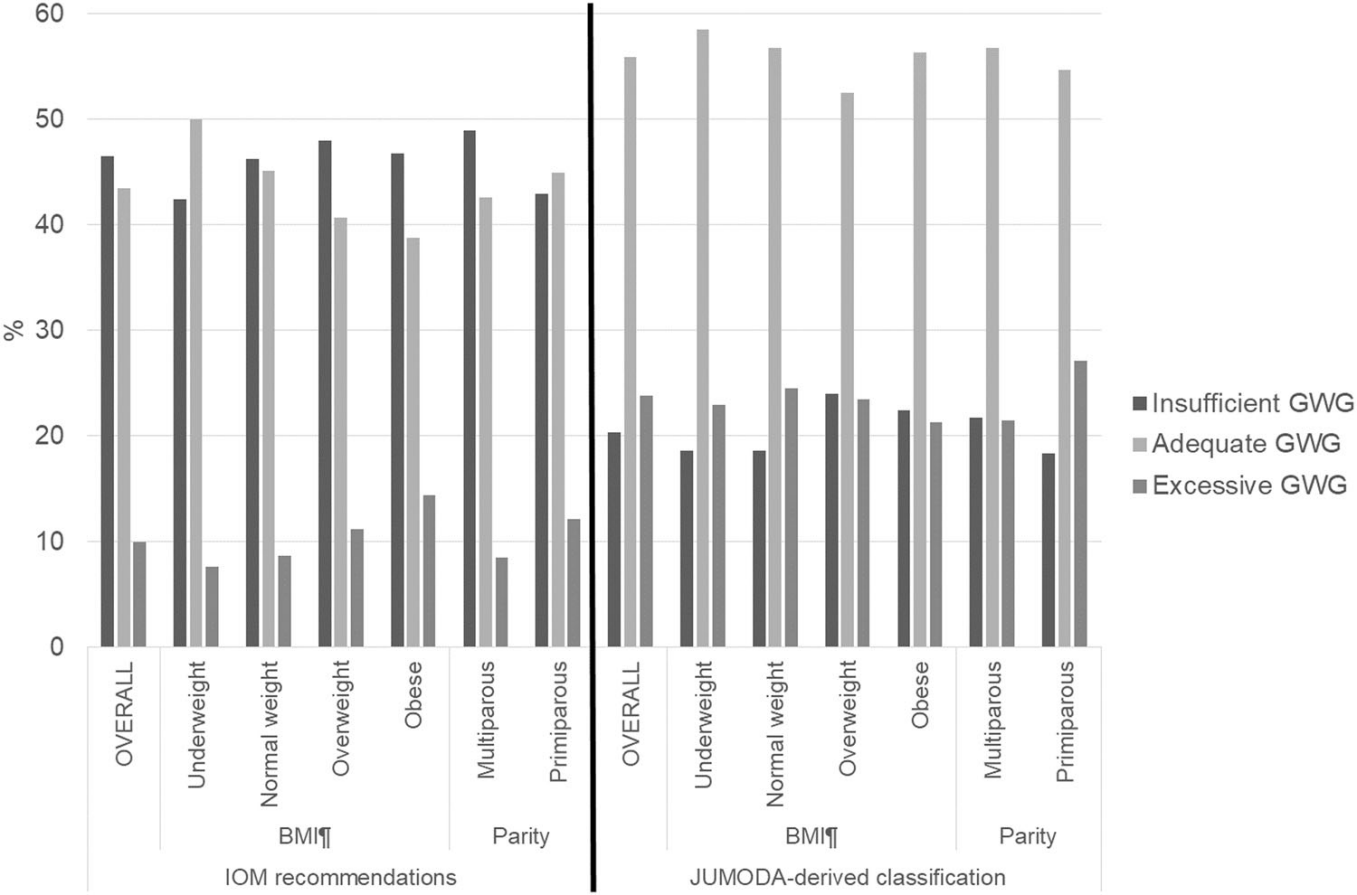
GWG adequacy in the JUmeaux MODe d'Accouchement (JUMODA) standard population according to the 2009 IOM recommendations and a JUMODA‐derived classification. For both classifications, GWG adequacy was defined based on the 25th and 75th percentiles of GWG within a standard population as: insufficient (below 25th percentile), adequate (25th–75th percentile), or excessive (above 75th percentile). The IOM recommendations were derived from a historical US cohort of women giving birth at 37–42 weeks with mean twin birthweight ≥2500 *g* (1979–1999) and are: normal weight: 16.8–24.5 kg (also utilized for underweight women as separate recommendation not provided); overweight: 14.1–22.7 kg; obese: 11.4–19.1 kg. The JUMODA‐derived classification was derived from the JUMODA standard population of term (37–42 weeks gestational age) twin livebirths with mean twin birthweight ≥2500 *g* (2014–2015) and are; underweight: 13–21 kg; normal weight: 13–20 kg; overweight: 11–19 kg; obese: 7–16 kg. ^¶^BMI (kg/m^2^): underweight: <18.5; normal weight: 18.5–24.9; overweight: 25–29.9; obese: ≥30. BMI, body mass index (prepregnancy); GWG, gestational weight gain; IOM, Institute of Medicine.

Based on IOM recommendations, almost half of women comprising the JUMODA standard population were classified as having insufficient GWG (46.5%), while few were classified as having excessive GWG (10.0%; Figure 2). Results were similar after stratification by maternal pregnancy BMI and parity.

## 4 DISCUSSION

GWG was lower in our standard population of twin gestations than in the cohort used to derive the IOM recommendations. Therefore, utilizing IOM methodology to classify GWG adequacy based on 25th and 75th percentiles of GWG in the JUMODA standard population, lower GWG was considered adequate. Additionally, the IOM recommendations classified almost half of women in the JUMODA standard population as having insufficient GWG and very few as having excessive GWG.

In line with our overall results, previous cohort studies with serial measures of GWG in twin gestations have shown increasing GWG curves over pregnancy (Hutcheon et al., 2018; Luke et al., 2003). The differences by prepregnancy BMI in the association between GWG and GA in our stratified analysis are intriguing and may point to the delicate physiologic balance between promotion of foetal growth (a component of GWG) versus maturity (Blickstein, 2002) and the need to meet nutritional demands while avoiding adverse effects of excess GWG, particularly given the inverse association found among women with underweight prepregnancy BMI who gain the most weight and may also be less able to sustain its incumbent physical demands. Given our small sample sizes after 39 weeks in this stratified analysis, additional prospective research in larger samples is needed confirm our results and to understand underlying physiology.

GWG was lower in our cohort in comparison to the IOM cohort, though our estimates increased slightly after adjusting for confounders, which may be due to differences both in the United States and French obstetric populations broadly and in the two standard twin cohorts specifically. As noted previously, the French obstetric population has a healthier BMI profile than the USA obstetric population (Coulm et al., 2017; Deputy et al., 2018), which could reflect cultural differences which also promote lower GWG, though lower GWG was observed in our cohort regardless of BMI. The difference in the temporality (IOM: 1979–1999; JUMODA: 2014–2015) could also explain results, as obstetric practices have changed greatly over the past decades. Our study was conducted after the publication of the IOM guidelines, which may have impacted the advice women received in prenatal visits. Indeed, current French recommendations for GWG for women with twin gestations and prepregnancy BMI 19–25 kg/m^2^ are 16–24 kg, in line with the current IOM recommendations (Haute Autorité de Santé, 2009), though specific recommendations are not provided for other BMI groups.

Notably, the application of IOM GWG guidelines for singletons in France did not produce such different results (26.8% insufficient GWG; 37.0% adequate GWG; 36.1% excessive GWG [Amyx et al., 2021]). However, the derivation of the IOM singleton guidelines differed substantially in that it was based on GWG associated with adverse outcomes rather than percentiles of GWG and included research commissioned in multiple singleton cohorts (2 USA, 1 Danish; dates ranging from 1988 to 2003) in addition to published research (Rasmussen & Yaktine, 2009).

Given the limited information provided in the IOM guidelines, direct comparison of the characteristics of the specific cohorts used to derive their guidelines and our JUMODA standard population is difficult. Based on information regarding the larger cohort (*n* = 2324 women with nonanomalous liveborn twins ≥28 weeks gestation) from which the IOM sample was drawn (Luke et al., 2003), 30% were included in their standard population, similar to the 29% in our study. However, these proportions are not directly comparable due to differences in the initial cohorts, in particular the GA at delivery and exclusion of underweight women from their standard population. The initial IOM cohort (37% White, non‐Hispanic; 19% Hispanic; 43% African American; 1% Asian [Luke et al., 2003]) was more racially/ethnically diverse the JUMODA standard population included in this analysis and the full JUMODA cohort (country of birth: 82% Europe; 11% North Africa; 6% sub‐Saharan Africa; 2% other [Korb et al., 2020]) though composition of the IOM standard population is not reported and could differ from their initial cohort given racial/ethnic disparities in birth outcomes.

Previous studies have similarly applied the IOM recommendations or derived alternate cutpoints to define GWG adequacy in twin gestations. More specifically, using similar methodology to ours, two studies in North American twin cohorts (single‐centre USA cohort, 2000–2017 [*n* = 247; Lipworth et al., 2021]; population‐based Canadian cohort, 2003–2014 [*n* = 326; Lutsiv et al., 2017]) found percentiles generally similar to the IOM guidelines (as did two additional USA studies with slightly different inclusion criteria [Hutcheon et al., 2018; Lin et al., 2022]), though trends of somewhat lower GWG percentiles for obese women (Lipworth et al., 2021; Lutsiv et al., 2017) and slightly higher GWG percentiles for women with normal and overweight BMI were observed (Lutsiv et al., 2017). In contrast, previous studies in non‐North American twin cohorts found results more similar to ours (multi‐centre Japanese cohort, 2013–2017 [*n* = 3936; Obata et al., 2021]; population‐based Chinese cohort using Chinese BMI standards, 2011–2017 [*n* = 6925; Chen et al., 2018]), finding lower 25th percentiles of GWG, though the 75th percentiles in the Chinese study varied in relation to the IOM guidelines.

Investigating the application of the IOM guidelines to define GWG adequacy in twin gestations, a systematic review found that, overall, 35.4% (95% confidence interval [CI] 30.0–41.1%, 13 studies) of women had insufficient and 21.4% (95% CI 14.2–29.5%, 9 studies) had excessive GWG (Lipworth et al., 2022). However, in examining individual studies, differences in the percentages were noted between North American and non‐North American populations. In North American twin cohorts of more general obstetric populations (two population‐based within a Canadian province [Lutsiv et al., 2017] or USA state [Gavard & Artal, 2014]; one using Consortium on Safe Labour data [Lal & Kominiarek, 2015]; three single‐centre [Fox et al., 2011; Lipworth et al., 2021; Liu et al., 2021]), the proportion of women with insufficient (27%–40.8%), adequate (32.5%–53%), or excessive GWG (15.6%–30.3%) was more balanced than in our French cohort. In the non‐North American standard twin populations mentioned above, an even more extreme shift in GWG adequacy classification was evident in Japan (80% insufficient; 19% adequate; 1% excessive [Obata et al., 2021]), though no strong shift was noted in the Chinese study (33% insufficient; 45% adequate; 22% excessive [Chen et al., 2018]). Evaluating general non‐North American obstetric populations, a single‐centre French study of liveborn twins delivered after 23 weeks 6 days (*n* = 878) classified a majority of women (53.3%) as having insufficient and few (7.3%) as having excessive GWG (Pécheux et al., 2019), with similar results in some other non‐North American twin cohorts, including single‐centre studies in Italy (52% insufficient; 42% adequate; 6% excessive [Algeri et al., 2018]), South Korea (51% insufficient; 40% adequate; 9% excessive [Choi et al., 2020]), and China (41% insufficient; 49% adequate; 10% excessive [Wang et al., 2018]). However, other results from Chinese (15% insufficient; 41% adequate; 44% excessive [Lin et al., 2019]) and Japanese twin cohorts (85% insufficient; 15% adequate [Shimura et al., 2021]) were mixed.

The differences noted between these studies and ours could be due study locations, as the IOM noted that their guidelines were specifically intended for USA populations (Rasmussen & Yaktine, 2009), suggesting the provisional IOM recommendations may indeed be applicable in North American obstetric populations.

Regardless of the differences noted above, studies have consistently found that inadequate GWG based on the IOM recommendations for twin gestations was associated with adverse outcomes. In North American twin cohorts, inadequate GWG based on the IOM recommendations was associated with adverse outcomes, in particular birthweight outcomes (Fox et al., 2011; Gavard & Artal, 2014; Lal & Kominiarek, 2015; Lin et al., 2022; Lipworth et al., 2021; Liu et al., 2021; Lutsiv et al., 2017). Similiarly, except in one Japanese study (Shimura et al., 2021), inadequate GWG based on IOM recommendations was nonetheless associated with adverse outcomes in non‐North American twin cohorts (Algeri et al., 2018; Chen et al., 2018; Choi et al., 2020; Lin et al., 2019; Wang et al., 2018), including the French study which found adequate GWG was associated with improvements in maternal and neonatal outcomes (Pre‐eclampsia, preterm delivery, low birthweight, respiratory distress, APGAR < 7 at 5 min [Pécheux et al., 2019])

### 4.1 Strengths

JUMODA provides extensive, rigorously collected data on a large, contemporary cohort of French twin pregnancies. Because the standard population utilized for our main analysis was limited to term deliveries, potential bias due to the inherent correlation between GWG and length of gestation was minimized. Given the provisional nature of the IOM recommendations for twin pregnancies, our study provides updated information to guide recommendations within a larger, more contemporary cohort, including for underweight women. Additionally, as previous studies which utilized the IOM methodology to derive percentiles were conducted in North American or Asian populations, our study provides results are more applicable to French and potentially also European populations. Though we note limitations of the IOM recommendations, in this initial analysis, their utilization is beneficial to allow consistent comparison across populations. No differences in maternal or pregnancy characteristics between eligible women included in the standard population and women excluded for missing data were noted (data not shown).

### 4.2 Limitations

Although JUMODA is a large cohort, few observations were available at extremes of GA at delivery and for underweight women, though we nonetheless had a higher number of women in each BMI group than were in the IOM cohort (normal weight BMI: 1534 vs. 409; overweight BMI: 562 vs. 154; obese BMI: 348 vs. 143) and all but one of the similar population‐based studies. Differences in the measurements utilized to calculate GWG could partially explain the differences between the IOM recommendations and JUMODA estimates. Though both studies utilized chart abstraction to obtain prepregnancy weight, in JUMODA, the initial weight measurement was from the beginning of pregnancy, while in the IOM cohort, pregravid weight was utilized. Further, in JUMODA, exact timing of the last prenatal visit is unknown (though given the frequency of prenatal visits at term for twin pregnancies in France, delivery weight was likely measured within a week of delivery), while in the USA cohort utilized for the IOM recommendations, GWG at delivery was measured within a week of delivery. Thus, GWG in the JUMODA cohort may be somewhat underestimated. Given the limited data related to characteristics of the cohort used to create the IOM recommendations, potential differences (beyond time period and location) between study populations are difficult to assess and therefore it is unclear whether the differences noted are due to differences in the study populations or the inadequacy of the IOM guidelines. Similarly, while we adjusted for the same covariates to determine adjusted means, it is possible our definitions for these variables were not identical to those used by the IOM, as they were not explicitly defined in their methods. Finally, given the complexities of incorporating these analyses, we did not include outcome data (to create outcome‐based recommendations or to evaluate the performance of the IOM recommendations and JUMODA classification). Therefore, it is unclear whether the IOM recommendations or JUMODA‐derived adequacy classification may be more clinically useful. To appropriately assess outcomes, careful consideration of which outcomes to evaluate, appropriate confounders to control for in adjusted analyses (given the variation in mechanisms underlying the association between insufficient/excessive GWG and different outcomes), and the correct study population (a more general population rather than the standard population would be necessary, in particular to evaluate preterm delivery or small for GA/large for GA) would be essential and better addressed in future research.

## 5 CONCLUSION

Using IOM recommendations to define GWG adequacy in our French cohort classified almost half of the women as having insufficient and a relatively low percentage as having excessive GWG. As we did not assess outcomes, it is unclear whether the IOM recommendations or the JUMODA‐derived classification is more appropriate for French twin gestations. Additional research in large, population‐based contemporary cohorts with prospective GWG assessment and assessment of adverse outcomes is needed to determine evidence‐based recommendations for optimal GWG in twin pregnancies.

## AUTHOR CONTRIBUTIONS

Thomas Schmitz contributed substantially to the design, data acquisition, and project administration of the JUMODA study. Melissa Amyx, Camille Le Ray, Thomas Schmitz, Jennifer Zeitlin, and Diane Korb contributed substantially to the design of the current study. Melissa Amyx conducted data analysis, interpreted the results, and developed the draft manuscript under the supervision of Camille Le Ray with input from Jennifer Zeitlin and Thomas Schmitz. All authors critically reviewed and approved the final manuscript.

## CONFLICT OF INTEREST

The authors declare no conflict of interest.

## Supporting information

Supporting information.

## Data Availability

The data set analyzed is not publicly available.

## References

[mcn13436-bib-0001] Algeri, P. , Pelizzoni, F. , Bernasconi, D. P. , Russo, F. , Incerti, M. , Cozzolino, S. , Mastrolia, S. A. , & Vergani, P. (2018). Influence of weight gain, according to Institute of Medicine 2009 recommendation, on spontaneous preterm delivery in twin pregnancies. BMC Pregnancy and Childbirth, 18(1), 6. 10.1186/s12884-017-1645-5 29298662 PMC5751880

[mcn13436-bib-0002] Amyx, M. , Zeitlin, J. , Hermann, M. , Castetbon, K. , Blondel, B. , & Le Ray, C. (2021). Maternal characteristics associated with gestational weight gain in France: A population‐based, nationally representative study. BMJ Open, 11(7), e049497. 10.1136/bmjopen-2021-049497 PMC8256790 34215613

[mcn13436-bib-0003] Blickstein, I. (2002). Normal and abnormal growth of multiples. Seminars in Neonatology, 7(3), 177–185. 10.1053/siny.2002.0105 12234742

[mcn13436-bib-0004] Bodnar, L. M. , Pugh, S. J. , Abrams, B. , Himes, K. P. , & Hutcheon, J. A. (2014). Gestational weight gain in twin pregnancies and maternal and child health: A systematic review. Journal of Perinatology, 34(4), 252–263. 10.1038/jp.2013.177 24457254 PMC4046859

[mcn13436-bib-0005] Chen, Y. , Liu, Y. , Zhang, Y. , Hu, R. , Qian, Z. , Xian, H. , Vaughn, M. G. , Liu, M. , Cao, S. , Gan, Y. , & Zhang, B. (2018). Gestational weight gain per pre‐pregnancy body mass index and birth weight in twin pregnancies: A cohort study in Wuhan, China. Scientific Reports, 8(1), 12496. 10.1038/s41598-018-29774-z 30131497 PMC6104075

[mcn13436-bib-0006] Choi, B. Y. , Hong, S. , Jeon, M. , Park, J. Y. , Oh, K. J. , & Hong, J. S. (2020). Gestational weight gain in twin pregnancies in Korea: Application of the 2009 Institute of Medicine recommendations. Obstetrics & Gynecology Science, 63(6), 690–699. 10.5468/ogs.20133 33137865 PMC7677065

[mcn13436-bib-0007] Coulm, B. , Bonnet, C. , & Blondel, B. (2017). French National Perinatal Survey 2016: Situation in 2016 and trends since 2010. INSERM. http://www.xn--epop-inserm-ebb.fr/en/grandes-enquetes/enquetes-nationales-perinatales 10.1016/j.jogoh.2017.09.002 29031048

[mcn13436-bib-0008] Deputy, N. P. , Dub, B. , & Sharma, A. J. (2018). Prevalence and trends in prepregnancy normal weight—48 states, New York City, and District of Columbia, 2011–2015. MMWR. Morbidity and Mortality Weekly Report, 66(51–52), 1402–1407. 10.15585/mmwr.mm665152a3 29300720 PMC5758298

[mcn13436-bib-0009] Fox, N. S. , Saltzman, D. H. , Kurtz, H. , & Rebarber, A. (2011). Excessive weight gain in term twin pregnancies: Examining the 2009 Institute of Medicine definitions. Obstetrics & Gynecology, 118(5), 1000–1004. 10.1097/AOG.0b013e318232125d 22015867

[mcn13436-bib-0010] Gavard, J. A. , & Artal, R. (2014). Gestational weight gain and maternal and neonatal outcomes in term twin pregnancies in obese women. Twin Research and Human Genetics, 17(2), 127–133. 10.1017/thg.2013.91 24423582

[mcn13436-bib-0011] Haute Autorité de Santé . (2009). Guidelines. [Texte des recommandations]. Journal De Gynecologie, Obstetrique Et Biologie De La Reproduction, 38(8 Suppl), S128–S131. 23477000

[mcn13436-bib-0012] Hutcheon, J. A. , Platt, R. W. , Abrams, B. , Braxter, B. J. , Eckhardt, C. L. , Himes, K. P. , & Bodnar, L. M. (2018). Pregnancy weight gain by gestational age in women with uncomplicated dichorionic twin pregnancies. Paediatric and Perinatal Epidemiology, 32(2), 172–180. 10.1111/ppe.12446 29378084 PMC5902642

[mcn13436-bib-0013] Korb, D. , Schmitz, T. , Seco, A. , Goffinet, F. , Deneux‐Tharaux, C. , & JUmeaux MODe d'Accouchement (JUMODA) Study Group and the Groupe de Recherche en Obstétrique et Gynécologie (GROG) . (2020). Risk factors and high‐risk subgroups of severe acute maternal morbidity in twin pregnancy: A population‐based study. PLoS One, 15(2):e0229612. 10.1371/journal.pone.0229612 32109258 PMC7048407

[mcn13436-bib-0014] Lal, A. K. , & Kominiarek, M. A. (2015). Weight gain in twin gestations: Are the Institute of Medicine guidelines optimal for neonatal outcomes? Journal of Perinatology, 35(6), 405–410. 10.1038/jp.2014.237 25634520 PMC4486049

[mcn13436-bib-0015] Lin, D. , Fan, D. , Wu, S. , Chen, G. , Li, P. , Ma, H. , Ye, S. , Rao, J. , Zhang, H. , Zeng, M. , Liu, Y. , Guo, X. , & Liu, Z. (2019). The effect of gestational weight gain on perinatal outcomes among Chinese twin gestations based on Institute of Medicine guidelines. BMC Pregnancy and Childbirth, 19(1), 262–019‐2411‐7. 10.1186/s12884-019-2411-7 31340779 PMC6657175

[mcn13436-bib-0016] Lin, D. , Huang, X. , Fan, D. , Chen, G. , Li, P. , Rao, J. , Zhang, H. , Guo, X. , Luo, C. , & Liu, Z. (2022). Association of optimal gestational weight gain ranges with perinatal outcomes across body mass index categories in twin pregnancies. JAMA Network Open, 5(7), e2222537. 10.1001/jamanetworkopen.2022.22537 35852802 PMC9297120

[mcn13436-bib-0017] Lipworth, H. , Barrett, J. , Murphy, K. E. , Redelmeier, D. , & Melamed, N. (2022). Gestational weight gain in twin gestations and pregnancy outcomes: A systematic review and meta‐analysis. BJOG: An International Journal Of Obstetrics and Gynaecology, 129(6), 868–879. 10.1111/1471-0528.17011 34775675

[mcn13436-bib-0018] Lipworth, H. , Melamed, N. , Berger, H. , Geary, M. , McDonald, S. D. , Murray‐Davis, B. , Murphy, K. E. , Redelmeier, D. A. , Yoon, E. W. , Barrett, J. F. R. , & Ram, M. (2021). Maternal weight gain and pregnancy outcomes in twin gestations. American Journal of Obstetrics and Gynecology, 225(5), 532.e1–532.e12. 10.1016/j.ajog.2021.04.260 33984302

[mcn13436-bib-0019] Liu, L. Y. , Zafman, K. B. , & Fox, N. S. (2021). Weight gain and pregnancy outcomes in overweight or obese women with twin gestations. The Journal of Maternal‐fetal & Neonatal Medicine, 34(11), 1774–1779. 10.1080/14767058.2019.1648421 31379228

[mcn13436-bib-0020] Luke, B. , Hediger, M. L. , Nugent, C. , Newman, R. B. , Mauldin, J. G. , Witter, F. R. , & O'sullivan, M. J. (2003). Body mass index—Specific weight gains associated with optimal birth weights in twin pregnancies. The Journal of Reproductive Medicine, 48(4), 217–224. 12746982

[mcn13436-bib-0021] Lutsiv, O. , Hulman, A. , Woolcott, C. , Beyene, J. , Giglia, L. , Armson, B. A. , Dodds, L. , Neupane, B. , & McDonald, S. D. (2017). Examining the provisional guidelines for weight gain in twin pregnancies: A retrospective cohort study. BMC Pregnancy and Childbirth, 17(1), 330. 10.1186/s12884-017-1530-2 28962593 PMC5622523

[mcn13436-bib-0022] Obata, S. , Shimura, M. , Misumi, T. , Nakanishi, S. , Shindo, R. , Miyagi, E. , & Aoki, S. (2021). Weight gain during twin pregnancy with favorable pregnancy outcomes in Japan: A retrospective investigation for new criteria based on perinatal registry data. PLoS One, 16(7), e0253596. 10.1371/journal.pone.0253596 34214100 PMC8253415

[mcn13436-bib-0023] Pécheux, O. , Garabedian, C. , Drumez, E. , Mizrahi, S. , Cordiez, S. , Deltombe, S. , & Deruelle, P. (2019). Maternal and neonatal outcomes according to gestational weight gain in twin pregnancies: Are the Institute of Medicine guidelines associated with better outcomes? European Journal of Obstetrics & Gynecology and Reproductive Biology, 234, 190–194. 30710766 10.1016/j.ejogrb.2019.01.010

[mcn13436-bib-0024] Rasmussen, K. , Yaktine, A. , & Institute of Medicine and National Research Council . (2009). Weight gain during pregnancy: Reexamining the guidelines. National Academy Press. 20669500

[mcn13436-bib-0025] Schmitz, T. , Prunet, C. , Azria, E. , Bohec, C. , Bongain, A. , Chabanier, P. , Deruelle, P. , De Tayrac, R. , Dreyfus, M. , Dupont, C. , Gondry, J. , Graesslin, O. , Kayem, G. , Langer, B. , Marpeau, L. , Morel, O. , Parant, O. , Perrotin, F. , Pierre, F. , …JUmeaux MODe d'Accouchement (JUMODA) Study Group and the Groupe de Recherche en Obstetrique et Gynecologie (GROG) . (2017). Association between planned cesarean delivery and neonatal mortality and morbidity in twin pregnancies. Obstetrics & Gynecology, 129(6), 986–995. 10.1097/AOG.0000000000002048 28486364

[mcn13436-bib-0026] Shimura, M. , Obata, S. , Misumi, T. , Miyagi, E. , & Aoki, S. (2021). Are the Institute of Medicine guidelines for optimal gestational weight gain in twin pregnancies applicable to Japanese women? Journal of Obstetrics and Gynaecology Research, 47(1), 337–342. 10.1111/jog.14529 33051925

[mcn13436-bib-0027] Vayssière, C. , Benoist, G. , Blondel, B. , Deruelle, P. , Favre, R. , Gallot, D. , Lemery, D. , Picone, O. , Pons, J. C. , Puech, F. , Quarello, E. , Salomon, L. , Schmitz, T. , Senat, M. V. , Sentilhes, L. , Simon, A. , Stirneman, J. , Vendittelli, F. , Winer, N. , Ville, Y. , & French College of Gynaecologists and Obstetricians . (2011). Twin pregnancies: Guidelines for clinical practice from the French College of Gynaecologists and Obstetricians (CNGOF). European Journal of Obstetrics & Gynecology and Reproductive Biology, 156(1), 12–17. 10.1016/j.ejogrb.2010.12.045 21277672

[mcn13436-bib-0028] Wang, L. , Wen, L. , Zheng, Y. , Zhou, W. , Mei, L. , Li, H. , Tong, C. , Qi, H. , & Baker, P. N. (2018). Association between gestational weight gain and pregnancy complications or adverse delivery outcomes in Chinese Han dichorionic twin pregnancies: Validation of the Institute of Medicine (IOM) 2009 guidelines. Medical Science Monitor, 24, 8342–8347. 10.12659/MSM.911784 30453309 PMC6256840

[mcn13436-bib-0029] Widen, E. M. (2018). Weight gain for gestational‐age charts in dichorionic twins: Tool for establishing optimal weight gain in twin pregnancies. Paediatric and Perinatal Epidemiology, 32(2), 181–183. 10.1111/ppe.12460 29508907 PMC5902643

[mcn13436-bib-0030] Zhong, W. , Fan, X. , Hu, F. , Chen, M. , & Zeng, F. (2021). Gestational weight gain and its effects on maternal and neonatal outcome in women with twin pregnancies: A systematic review and meta‐analysis. Frontiers in Pediatrics, 9, 1‐12. 10.3389/fped.2021.674414 PMC8298912 34307252

