# Supplementary material for: Gestational weight gain adequacy among twin pregnancies in France
Source: Matern Child Nutr. 2022 Oct 12;19(1):e13436. doi: 10.1111/mcn.13436 (PMC9749591; doi:10.1111/mcn.13436)
Supplement: Supplementary file 1 — Supporting information. [file MCN-19-e13436-s001.pdf]

Supplemental Figure 1: Participant flowchart

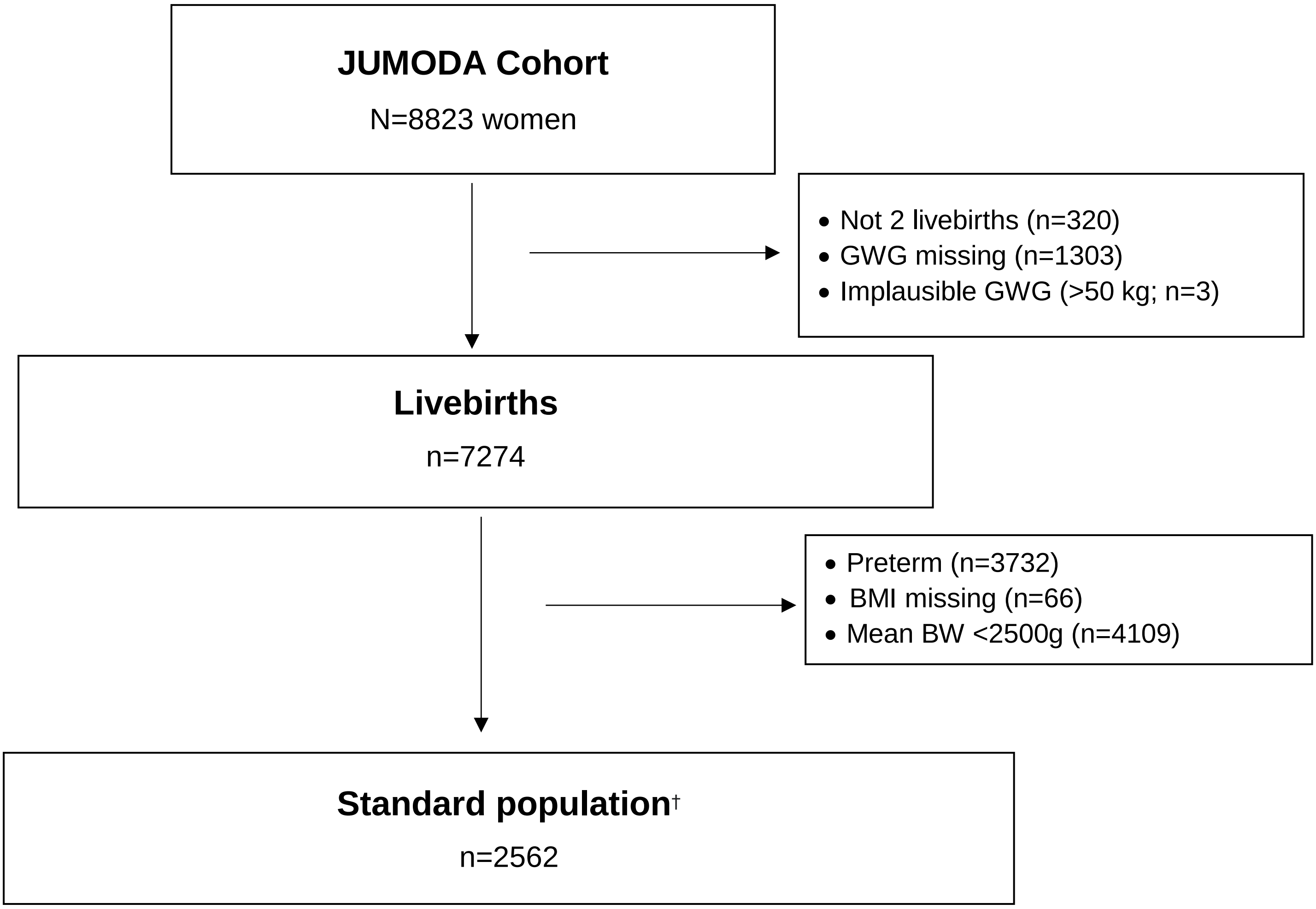


†Term (37-42 weeks gestational age) twin livebirths with optimal BW (mean twin BW ≥2500)

Abbreviations: BMI, body mass index; BW, birthweight; GWG, gestational weight gain
